# Editorial: Decoding muscle adaptation through skeletal muscle negative data: understanding the signaling factors involved

**DOI:** 10.3389/fphys.2024.1440736

**Published:** 2024-06-20

**Authors:** Marshall A. Naimo, Brandon M. Roberts, Stephen E. Alway

**Affiliations:** ^1^ Military Performance Division, US Army Research Institute of Environmental Medicine, Natick, MA, United States; ^2^ Laboratory of Muscle Biology and Sarcopenia, Division of Regenerative and Rehabilitation Sciences, College of Health Professions, University of Tennessee Health Science Center, Memphis, TN, United States; ^3^ Center for Muscle, Metabolism, and Neuropathology, Division of Regenerative and Rehabilitation Sciences, College of Health Professions, University of Tennessee Health Science Center, Memphis, TN, United States; ^4^ Department of Physiology, College of Medicine, University of Tennessee Health Science Center, Memphis, TN, United States; ^5^ The Tennessee Institute of Regenerative Medicine, Memphis, TN, United States

**Keywords:** exercise, negative data, skeletal muscle quality, pathways, adaptive response, overload, biomarkers, mechanisms and models

Skeletal muscle adapts to exercise by improving metabolism and structure, leading to elevated performance. The identification of multiple signaling pathways that contribute to muscle adaptation including, but not limited to, JAK2/STAT3 (Yao et al., 2024), TGFβ-Smad2/3-ATF4 (Vanhoutte et al., 2024), Hippo/YAP (Pan et al., 2024), Ca (2+)-NFATC1 (Zhou et al., 2024a), PROKR1-CREB-NR4A2 (Mok et al., 2024), mitochondria cross talk (Reisman et al., 2024), mitochondria biogenesis and function (Mesquita et al., 2021; Marzetti et al., 2024), redox levels (Zhou et al., 2024b), and autophagy (Parousis et al., 2018), and also individual mediators like GADD42a (Marcotte et al., 2023), Trim63 (da Mata et al., 2024), mTORC1 (DHulst et al., 2022; McIntosh et al., 2023), YAP (Brooks et al., 2018) and AMPK (Kido et al., 2023; Roberts et al., 2024) have provided important insight into our understanding of muscle plasticity. However, signaling proteins or pathways that do not change with exercise are rarely reported and often interpreted as unimportant and therefore are not published. Nevertheless, this information could provide critical insight into molecular targets for adaptation to occur. Furthermore, exercise-induced physiological changes require concerted interactions from multiple signaling cascades in different organ systems simultaneously. Thus, negative results (e.g., proteins or molecules that do not change) can highlight which pathways and molecules may not be involved. Reporting molecular signals that do not change significantly in response to exercise could reduce lost time and resources conducting experiments to test proteins that do not change. Thus, results from both positive and negative signaling are important for identifying novel biomarkers and understanding the adaptive exercise responses, including performance, hypertrophy, aerobic/anaerobic capacity, and muscle quality. Thus, the aim of this Research Topic was to provide a platform to publish negative results that were discovered through well-designed research trials on exercise training adaptations and related signaling mechanisms. Manuscripts considered for this Research Topic met all the requirements for submission, including providing details on the methods, a careful description of the controls for the experiments, a critical evaluation of the results and interpretation of the data, including a rationale/hypothesis for the importance of the negative results to the field, and suggestions for future directions to address the issue.

The five articles in this Research Topic covered different aspects of exercise, from aerobic exercise to resistance exercise. Halle et al. used a mouse model of long-duration treadmill running to investigate the effects FOLFOX chemotherapy, a commonly used approach to treat colorectal cancer, on the adaptations to short or long-duration treadmill exercise training. The results showed that while FOLFOX attenuated early exercise responses of osteocalcin, LIF, and IL-6, it did not change plantaris muscle COXIV activity and plasma levels of adiponectin. These findings highlight the need for more evidence-based exercise prescription guidelines for cancer survivors following chemotherapy and understanding how signaling mechanisms to exercise differ from normal individuals not undergoing chemotherapy. In a progressive resistance treadmill protocol, Yee et al. examined the effects of 12-week of treadmill training on muscle cellular content and transcriptional changes, along with whole body and muscle function, following recovery from disuse atrophy. The authors showed increases in exercise capacity, physical function, endothelial function, and a decrease in fat mass. However, there was no change in mRNAs for tibialis anterior muscle atrophy, pro-inflammatory, anti-inflammatory, and metabolism, or the content of macrophages, satellite cells, capillaries and collagen, with only a trend for increased tibialis anterior muscle mass during recovery from disuse atrophy. Collectively, the authors data suggests that progressive treadmill training in aged male mice had minor effects on muscle remodeling and growth following disuse atrophy. As these findings show different adaptative responses than reported in young animals, the data indicate the need for additional future research to identify the underlying signaling mechanisms that are required for aged muscle to recover following disuse atrophy. The third article in the Research Topic, from Burke et al., used a barium chloride-induced muscle damage model in adult mice. They reported minimal effects of Apolipoprotein E (ApoE), which included ApoE2, ApoE3, and ApoE4, on muscle regeneration following an extreme muscle damage model. The authors acknowledged the limitation that their injury model is a non-physiological model of muscle regeneration and that more studies are needed to determine if similar results hold true following adaptation to exercise training.

Next, Mattingly et al. used both a synergistic ablation model to induce hypertrophy of the plantaris muscle adult mice and a 6-week resistance training program in college-aged males to examine muscle protein lactylation responses. The authors found that there were no changes in lactylation-dependent mRNA in human biopsy muscle samples obtained following resistance training, and similarly there were no differences in lactylation content in ablation-induced overload muscle versus control. These findings challenge the long-held dogma that lactate has an impactful role in muscle growth as part of the adaptive response to resistance training. The final article in the Research Topic was a systematic review performed by Ye et al. aimed to investigate 8-OHdG as an indicator of DNA damage after an acute bout of exercise in trained and untrained adults. The results demonstrated that while there was a medium effect size of resistance exercise causing increased levels of circulating 8-OHdG for both trained and untrained adults, this effect only held true for untrained individuals following aerobic exercise. However, like resistance exercise, high-intensity aerobic exercise significantly increased circulating 8-OHdG. These results showed that 8-OHdG is dependent on exercise modality and training status, and that the role of 8-OHdG in the skeletal muscle inflammatory response following an acute bout of exercise needs further investigation.

In conclusion, the articles comprising this Research Topic have identified important molecules that are not a part of the underlying musculoskeletal signaling cascades that contribute to the adaptive response to exercise under different homeostatic conditions. The intent is that other researchers can use the information gathered from these experiments to refine their research and increase their chances of making new discoveries in the pursuit of characterizing the signaling mechanisms that contribute to the adaptive response to exercise more completely.

## References

[B1] Brooks M. J. Hajira A. Mohamed J. S. Alway S. E. (2018). Voluntary wheel running increases satellite cell abundance and improves recovery from disuse in gastrocnemius muscles from mice. J. Appl. Physiol. 124, 1616–1628. 10.1152/japplphysiol.00451.2017 29470148 PMC6032091

[B2] da Mata G. E. Bricola R. Ribeiro D. N. Simabuco F. M. Pauli J. R. de Freitas E. C. (2024). Acute exercise modulates Trim63 and Bmal1 in the skeletal muscle of IL-10 knockout mice. Cytokine 175, 156484. 10.1016/j.cyto.2023.156484 38159471

[B3] DHulst G. Masschelein E. De Bock K. (2022). Resistance exercise enhances long-term mTORC1 sensitivity to leucine. Mol. Metab. 66, 101615. 10.1016/j.molmet.2022.101615 36252815 PMC9626937

[B4] Kido K. Eskesen N. O. Henriksen N. S. Onslev J. Kristensen J. M. Larsen M. R. (2023). AMPKγ3 controls muscle glucose uptake in recovery from exercise to recapture energy stores. Diabetes 72, 1397–1408. 10.2337/db23-0358 37506328 PMC10545559

[B5] Marcotte G. R. Miller M. J. Kunz H. E. Ryan Z. C. Strub M. D. Vanderboom P. M. (2023). GADD45A is a mediator of mitochondrial loss, atrophy, and weakness in skeletal muscle. JCI Insight 8, e171772. 10.1172/jci.insight.171772 37815864 PMC10721312

[B6] Marzetti E. Calvani R. Coelho-Junior H. J. Landi F. Picca A. (2024). Mitochondrial quantity and quality in age-related sarcopenia. Int. J. Mol. Sci. 25, 2052. 10.3390/ijms25042052 38396729 PMC10889427

[B7] McIntosh M. C. Sexton C. L. Godwin J. S. Ruple B. A. Michel J. M. Plotkin D. L. (2023). Different resistance exercise loading paradigms similarly affect skeletal muscle gene expression patterns of myostatin-related targets and mTORC1 signaling markers. Cells 12, 898. 10.3390/cells12060898 36980239 PMC10047349

[B8] Mesquita P. H. C. Vann C. G. Phillips S. M. McKendry J. Young K. C. Kavazis A. N. (2021). Skeletal muscle ribosome and mitochondrial biogenesis in response to different exercise training modalities. Front. Physiol. 12, 725866. 10.3389/fphys.2021.725866 34646153 PMC8504538

[B9] Mok J. Park J. H. Yeom S. C. Park J. (2024). PROKR1-CREB-NR4A2 axis for oxidative muscle fiber specification and improvement of metabolic function. Proc. Natl. Acad. Sci. U. S. A. 121, e2308960121. 10.1073/pnas.2308960121 38232288 PMC10823220

[B10] Pan C. Hao X. Deng X. Lu F. Liu J. Hou W. (2024). The roles of Hippo/YAP signaling pathway in physical therapy. Cell Death Discov. 10, 197. 10.1038/s41420-024-01972-x 38670949 PMC11053014

[B11] Parousis A. Carter H. N. Tran C. Erlich A. T. Mesbah Moosavi Z. S. Pauly M. (2018). Contractile activity attenuates autophagy suppression and reverses mitochondrial defects in skeletal muscle cells. Autophagy 14, 1886–1897. 10.1080/15548627.2018.1491488 30078345 PMC6152519

[B12] Reisman E. G. Hawley J. A. Hoffman N. J. (2024). Exercise-regulated mitochondrial and nuclear signalling networks in skeletal muscle. Sports Med. 54, 1097–1119. 10.1007/s40279-024-02007-2 38528308 PMC11127882

[B13] Roberts B. M. Geddis A. V. Ciuciu A. Reynoso M. Mehta N. Varanoske A. N. (2024). Acetaminophen influences musculoskeletal signaling but not adaptations to endurance exercise training. FASEB J. 38, e23586. 10.1096/fj.202302642R 38568858

[B14] Vanhoutte D. Schips T. G. Minerath R. A. Huo J. Kavuri N. S. S. Prasad V. (2024). Thbs1 regulates skeletal muscle mass in a TGFβ-Smad2/3-ATF4-dependent manner. Cell Rep. 43, 114149. 10.1016/j.celrep.2024.114149 38678560 PMC11217783

[B15] Yao B. Li L. Guan X. Zhu J. Liu Q. Qu B. (2024). Endurance training inhibits the JAK2/STAT3 pathway to alleviate sarcopenia. Physiol. Res. 73, 295–304. 10.33549/physiolres.935234 38710060 PMC11081189

[B16] Zhou Y. Liu X. Qi Z. Huang C. Yang L. Lin D. (2024a). Lactate-induced metabolic remodeling and myofiber type transitions via activation of the Ca(2+)-NFATC1 signaling pathway. J. Cell Physiol. 10.1002/jcp.31290 38686599

[B17] Zhou Y. Zhang X. Baker J. S. Davison G. W. Yan X. (2024b). Redox signaling and skeletal muscle adaptation during aerobic exercise. iScience 27, 109643. 10.1016/j.isci.2024.109643 38650987 PMC11033207

